# ROCK1 reduces mitochondrial content and irisin production in muscle suppressing adipocyte browning and impairing insulin sensitivity

**DOI:** 10.1038/srep29669

**Published:** 2016-07-14

**Authors:** Xiaoshuang Zhou, Rongshan Li, Xinyan Liu, Lihua Wang, Peng Hui, Lawrence Chan, Pradip K. Saha, Zhaoyong Hu

**Affiliations:** 1 Nephrology Division, Shanxi Province People’s Hospital of Shanxi Medical University, Taiyuan, China; 2 Nephrology Division, Second Hospital of Shanxi Medical University, Taiyuan, China; 3 Nephrology Division, The third affiliated hospital of Sun Yat-sen University, Guangzhou, China; 4 Endocrinology Division, Department of Medicine, Baylor College of Medicine, Houston, Texas, USA; 5 Nephrology Division, Department of Medicine, Selzman Institute for Kidney Health, Baylor College of Medicine, Houston, Texas, USA

## Abstract

Irisin reportedly promotes the conversion of preadipocytes into “brown-like” adipocytes within subcutaneous white adipose tissue (WAT) via a mechanism that stimulates UCP-1 expression. An increase in plasma irisin has been associated with improved obesity and insulin resistance in mice with type 2 diabetes. But whether a low level of irisin stimulates the development of obesity has not been determined. In studying mice with muscle-specific constitutive ROCK1 activation (mCaROCK1), we found that irisin production was down-regulated and the mice developed obesity and insulin resistance. Therefore, we studied the effects of irisin deficiency on energy metabolism in mCaROCK1 mice. Constitutively activation of ROCK1 in muscle suppressed irisin expression in muscle resulting in a low level of irisin in circulation. Irisin deficiency reduced heat production and decreased the expression of uncoupling protein 1 (UCP1) in brown adipose tissue (BAT) and subcutaneous WAT. Moreover, mCaROCK1 mice also displayed impaired glucose tolerance. Notably, irisin replenishment in mCaROCK1 mice partially reversed insulin resistance and obesity and these changes were associated with increased expression of UCP1 and Pref-1 in subcutaneous WAT. These results demonstrate that irisin mediates muscle-adipose tissue communication and regulates energy and glucose homeostasis. Irisin administration can correct obesity and insulin resistance in mice.

Obesity and insulin resistance occur commonly and are major factors in the pathogenesis of type 2 diabetes and cardiovascular disorders^1^,^2^,^3^. Several factors contribute to the development of obesity, including increase in fatty acids in muscle, macrophage infiltration into adipose tissue, cytokine production an altered secretion of myokines^4^. White adipose tissue (WAT) stores excess energy as triglycerides while brown adipose tissue (BAT), recognized from an abundance of mitochondria and uncoupling protein-1 (UCP-1), decreases energy storage by generating heat (thermogenesis). Within WAT, there are precursor cells which can be converted into brown-like adipocytes. Therefore, promoting white adipocytes to transform brown-like adipocytes might limit WAT stores. Conversely, increasing the differentiation of preadipocytes into mature white adipocytes would increase adipose tissue accumulation resulting in obesity^5^,^6^. Although mechanisms by which preadipocytes differentiate into adipocytes have been intensively studied^7^, the influence of skeletal muscle on this process remains unclear.

It is known that physical activity influences energy metabolism. For example, even short periods of physical inactivity are associated with decreased insulin sensitivity, attenuation of postprandial lipid metabolism and the accumulation of visceral adipose tissue^8^,^9^. These reports suggest that skeletal muscle can interact with others organs to limit adipose tissue accumulation. Support for such an activity of skeletal muscle is that it is a secretory organ releasing humoral factors called “myokines”. For example, muscle produces myostatin which impairs lipid and glucose metabolism. Conversely, inhibition of myostatin improves insulin sensitivity and suppresses lipogenesis^10^,^11^. Irisin, a myokine produced in and released from skeletal muscle after exercise, reportedly acts to increase brown-like adipocytes within the subcutaneous white adipose tissue resulting in weight loss, enhanced energy expenditure in mice^12^,^13^,^14^,^15^,^16^. It is not known if suppression of irisin causes obesity and insulin resistance.

Skeletal muscle consists of slow-twitch, type I fibers and fast-twitch, type II fibers. Type I fibers have a high mitochondrial content and irisin is predominately produced in these fibers^17^. Mitochondrial dynamics in muscle is specialized according to myofiber types within skeletal muscle, regulating muscle function and homeostasis. Recent reports indicate that Rho-kinase-1 (ROCK1) can stimulate mitochondrial fission and increase autophagy activity in podocyte and HeLa cells^18^,^19^. ROCK1 (molecular weight ~160 kD) is a Ser/Thr protein kinase identified as a GTP-Rho-binding protein^20^,^21^. ROCK1 activity is enhanced when it binds to RhoA through a Rho-binding domain^21^. Alternatively, caspase-3 can cleave ROCK1 yielding a 130 kD, constitutively activate form of ROCK1 (CaROCK1)^22^. Activated ROCK1 affects many cellular processes, including mitochondrial fission, signal transduction and cytoskeletal organization^18^,^23^. We have generated a transgenic mouse with muscle-specific, constitutively active ROCK1 (mCaROCK1). These mice have a lower mitochondrial content in type I fibers and the mice develop obesity and impaired insulin sensitivity. To determine how mCaROCK1 affects phenotypic changes, we examined glucose homeostasis and insulin action in mice with constitutively activated ROCK1. We measured irisin production and examined whether a decrease in irisin results in obesity and insulin resistance.

## Materials and Methods

### Generation of mice with muscle-specific constitutively active ROCK1 activation

Experimental procedures conform to the Guide for the Care and Use of Laboratory Animals published by the US National Institutes of Health; all animal procedures were approved by the Baylor College of Medicine Institutional Animal Care and Use Committee (protocol AN-625, AN-3965). To generate mCaROCK1 mice, we bred MCK-Cre transgenic mice (Jackson laboratory, Bar Harbor, ME) with mice bearing the floxed, transcriptional stop cassette that is upstream of the constitutively active ROCK1 (Supplemental Fig. 1a)^18^,^24^. Mouse breeding strategy is shown in supplemental Fig. 1b. Muscle-specific ROCK1 activation was confirmed by ROCK activity assay (see below). For irisin treatment experiment, mCaROCK1 and control mice were given irisin (100 μg/Kg/day, ip) for 3 weeks. Irisin was purchased from (Cayman Chemical, Ann Arbor, MI). Mice were housed with a 12-h light-dark cycles and were given free access to food and water.

#### ROCK1 activity assay

ROCK1 activity is determined by measuring the phosphorylation of MYPT, a substrate of ROCK1^25^. Briefly, 20 mg of TA muscle or other tissues were lysed in RIPA buffer (containing 0,1 mM EGTA, 0,1 M DTT, 10 mM MgCl_2_). After centrifugation (13,200 × g), 500 ng of MYPT1 (amino acids 654-880, ~60 kD, EMD Millipore, Billerica, MA) and ATP (1 mM) were added followed by incubation for 30 min at 30 °C before the reaction was stopped by adding 2x SDS Sample loading Buffer. Western blots were performed using phospho-MYPT1 (Thr-850, EMD Millipore) at 1:500 dilutions.

#### mtDNA Content Quantification by real-time PCR

Mitochondrial DNA (mtDNA) was co-purified with genomic DNA from muscle tissues using the DNeasy kit (QIAGEN), Ct values determined for ND1 gene encoded by mtDNA and LPL gene encoded by the nuclear DNA, and the relative mtDNA copy number calculated by normalizing to genomic LPL gene copy number. Primer sequences for mND1 and LPL gene are provided below: mND1 (F 5′-CCCATTCGCGTTATTCTT-3′, R 5′-AAGTTGATCGTAACGGAAGC-3′); LPL gene (F 5′-GGATGGACGGTAAGAGTGATTC-3′; R 5′-ATCCAAGGGTAGCAGACAGGT-3′).

#### Blood glucose and GTT assay

Mice were fasted for 6 h and a glucose tolerance test (GTT) was performed by ip injection of D-glucose (1.0 g/mouse kg). Glucose in tail vein blood was measured at 0, 15, 30, 60 and 120 min using the Accu-CHEK Advantage blood glucose meter (Accu-CHEK; Indianapolis, IN).

#### Metabolic assay

All metabolic assays were performed by The Mouse Metabolism Core at Baylor College Medicine (www.bcm.edu/research/centers/diabetes-research/core-labs/mouse-metabolism-core). Briefly, the metabolic parameters were measured using the Comprehensive laboratory animal monitoring system (CLAMS™, Columbus Instruments, Columbus, OH). The instrument is a set of live-in cage for automated, non-invasive and simultaneous monitoring of feeding and drinking. 6-months-old wild-type and mCaROCK1 mice were individually placed in CLAMS cages and monitored for day-cycle and night-cycles during 72 h period. Food and water consumption are measured directly as accumulated data and expressed as g/day or ml/d. The cage also are equipped with a set of sensors to record hourly volume of oxygen consumed (VO2 ml/Kg/h), volume of carbon dioxide produced (VCO2 ml/Kg/h) and heat production (Kcal/h). Energy expenditure was normalized to lean body mass determined from body composition analysis.

#### Body composition analysis (MRI)

We used the 4 in1−900 model Echo Magnetic Resonance Body Composition Analyzer (Echo Medical system, Houston, TX) to assess the total fat mass in wild-type and mCaROCK1 mice according to manufacturer’s directions. This Nuclear Magnetic Resonance (NMR) systems can measure precisely whole body fat mass, lean tissue mass, free water, and total body water in live animals up to 100 grams, without the need for anesthesia. The system needs to calibrate using a measured amount of oil at 37 °C. After measuring the mouse body weight, each mouse is then placed inside a specific holder just fit for that mouse (less movement will provide more precise results) and then insert that holder to analyzer. The procedure requires 69 seconds per mouse. Data are presented in grams and total body adipose fat was factored for body weights in all animals and expressed as a percentage.

*Locomotion activity assay* was performed by The Mouse Metabolism Core at Baylor College Medicine using the VersaMax Animal Activity Monitoring System (AccuScan Instruments, Inc.; Columbus, OH). Mice were individually placed in chambers which are equipped with 48 horizontal and vertical infrared sensor beams to counter X-Y total activity (all horizontal beam breaks in counts), X-Y ambulatory activity (consecutive horizontal beam breaks) and Z activity (all vertical beam breaks). Mice were acclimated in the monitoring chambers for 1 day before the experiment. Results were collected every 5 min for each mouse over a period of 24 hr.

#### Biochemical assay

Plasma insulin was determined with an Ultra-Sensitive Mouse Insulin ELISA Kit (Crystal Chem, Inc.) following the manufacturer’s protocol. Plasma FFA was measured with a NEFA C Test Kit (Wako Chemicals, Richmond, VA). Plasma triglycerides were analyzed using plasma Triglyceride Determination Kit (Sigma-Aldrich, St, Louis MO) and plasma cholesterol was determined by a Cholesterol/Cholesteryl Ester Quantitation Kit (BioVision, Milpitas, California). Plasma IL-6 and myostatin levels were measured by using ELISA Kit (EMD Millipore). Plasma irisin was determined using a mouse irisin ELISA Kit (Phoenix pharmaceutical, Burlingame, CA).

#### Tissue immunohistostaining and electron microscopy

Inguinal white adipose tissues were collected and fixed with 10% formalin (Fisher, Pittsburgh, PA). After paraffin imbedding, tissue samples were sectioned (5-μm) and stained for UCP1 (Sigma-Aldrich) and Pref-1 (Cell Signaling Technology, Beverly, MA). Immunohischemistry was performed with avidin-biotin elite kit (Vector Laboratories, Burlingame, CA) following the manufacturer’s protocol. For electron microscopy, fresh isolated EDL and Soleus muscles for wild-type and mCaROCK1 mice were pinned on a small plastic board at rest position and followed a fix in 2% paraformal-dehyde +2.5% glutaraldehyde in 0.1 m sodium cacodylate buffer for overnight at 4 °C. Then, samples were incubated in 1% osmium tetroxide followed by 1% uranyl acetate for 1 h. After dehydration, samples were embedded in resin with alignment of the longitudinal axis of myofibers. Thin (80–100 nm) sections were cut using an Ultracut E ultramicrotome (Leica Microsystems, Bannockburn, IL). After stained with uranyl acetate/lead citrate, sections were examined and imaged at 80 keV using Hitachi H-7500 transmission electron microscopy.

#### Cell culture and transfection

C_2_C_12_ myoblasts (passage 7) were cultured in DMEM with 10% FBS (HyClone, Logan, UT), penicillin (200 units/ml), and streptomycin (50 μg/ml) (Life Technologies). Plasmid DNA (500 ng/ml) was introduced into cells (200,000/ml) using an Invitrogen electroporation transfection system (voltage: 1640v, three pulses with pulse width 10 ms).

#### Total RNA and quantitive Real-time PCR

Total RNA was extracted using TRIzol (Sigma-Aldrich) and precipitated in isopropanol. 0.5 μg of RNA from each sample was converted into cDNAs using iScript cDNA Synthesis Kit (Bio-Rad, Hercules, CA). SYBR Green Real-time quantitative PCR was performed using CFX96 System (Bio-Rad Laboratories). The threshold cycle (Ct) is defined as the number of cycles required for the fluorescence signal to exceed the detection threshold. mRNA expression was standardized to the ribosomal protein L39 (RPL39) gene^26^,^27^ or beta-actin, and mRNA expression was calculated as the difference between the threshold values of the two genes (2 − ΔCt). The primer sequences for mouse myokines are shown in Supplemental Fig. 3.

#### Antibodies and western blot analyses

Anti-Pref-1 (DLK-1), anti-SDHA and VDAC, anti-GAPDH were purchased form Cell Signaling Technology (Cambridge, MA). Anti-ROCK1 antibody and anti-tubulin antibodies were purchased from Santa Cruz bio-technology (Santa Cruz, CA). For western blotting, lysates of muscles (EDL, soleus or gastrocnemius) or C_2_C_12_ cells were prepared in RIPA buffer (20 mM Tris, pH 7.5, 5 mM EDTA, 150 mM NaCl, 1% NP40, 0.5% Na-deoxycholate, 0.025% SDS, 1 mM Na-orthovanadate, 10 mM NaF, 25 μM β-glycerophosphate) containing protease inhibitors (Roche, Indianapolis, IN). Adipose tissue lysates were prepared from ~200 mg of inguinal WAT or scapular BAT by homogenizing tissues in 1 ml RIPA buffer. After centrifugation at 13,200 × g for 15 min at 4 °C, the supernatants were subjected to western blotting as described^22^. GAPDH or tubulin was used as a loading control.

#### FNDC5 promoter assay

To construct an FNDC5 (Irisin precursor) promoter-reporter, we PCR-amplified a mouse FNDC5 promoter (−2000/+70) using a forward primer (5′-GGGGTACC GATAGCCAACCGAAGGAC-3′) and a reverse primer (5′-CCCAAGCTTAAGCAGACGCAGCCTAGC-3′). These were derived from NCBI Genome Annotation, NW_027402. The promoter fragment was subcloned into KpnI and HindIII sites of PGL3 plasmid (Promega, Madison, WI). To evaluate the regulation of FNDC5 transcription by constitutively active ROCK1, we co-transfected C_2_C_12_ cells with FNDC5-PGL3 plus an equal amount of pCAG-myc vector or a pCAG-myc vector that encodes constitutively active ROCK1 (CaROCK1) or a kinase-deficient mutant ROCK1 (mutROCK1)^28^. After 24 h, luciferase activities were measured using the Dual Luciferase Assay System (Promega, Madison, WI, USA) according to manufacturer’s protocol.

#### Statistical analysis

Results are presented as mean ± SEM. For experiments comparing two groups, we analyzed results by the Student-Newman-Keul’s, two-tail, unpaired tests. When more than 2 groups were compared, one-way ANOVA followed by Scheffe’s post hoc test were used to analyze difference between irisin-treated and nontreated group in mCaROCK1 mice. Differences were considered statistically significant at p < 0.05(*) or p < 0.01(**).

## Results

### Muscle-specific ROCK1 activation decreases mitochondrial content in type I myofibers

**Figure 1:**
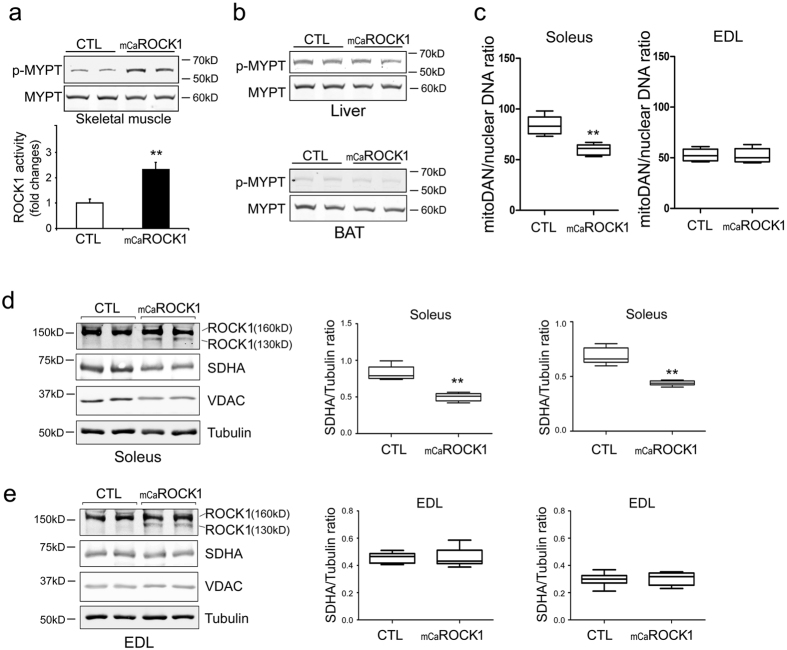
Muscle-specific CaROCK1 activation decrease mitochondrial content in type I myofibers. (**a**) In TA muscles of 4-months-old mCaROCK1 and littermate, control mice, ROCK1 activities were assessed using ROCK1 activity assay: MYPT1 peptide (60 kD, a substrate of ROCK1) was added into the muscle lysates and then incubated at 30 °C for 30 min. ROCK1 activity was assessed by quantifying the ratio of phosphorylated MYPT1 (p-MYPT1)/MYPT using western blots. Data are presented as the mean ± SEM, n = 5, *p < 0.05. (**b**) In liver and brown adipose tissue (BAT) of Control (CTL) and mCaR1 mice, ROCK1 activities were assessed by ROCK1 activity assay. (**c**) Relative mitochondrial content assessed by mitochondrial DNA (mDNA) copies numbers normalized with nuclear DNA (nDNA) copies number in soleus (Sol) and EDL extensor digitorum longus (EDL) skeletal muscles (mean ± SEM, n = 5, *p < 0.05). (**d**) The expressions of intact ROCK1 (160 kD) and constitutive active ROOCK1(CaROCK1, 130 kD) or mitochondrial specific protein (SDHA and VDAC) were examined in Soleus muscles of Control and mCaROCK1 mice by western blots. Tubulin was as loading control (left panel). Quantitative analysis of western blots (middle and right panel) (mean ± SEM, n = 5, **p < 0.01). (**e**) ROCK1 and CaROCK1 or mitochondrial specific protein (SDHA and VDAC) were examined in EDL muscles of Control and mCaROCK1 mice by western blots (left panel). Quantitative analysis of western blots (middle and right panel) (mean ± SEM, n = 5).

The mCaROCK1 mice exhibited no growth abnormalities until 4 months of age and their birth were in the expected Mendelian ratios. ROCK1 activity in tibialis anterior (TA) muscles was 2.3-folds higher than the values in muscle of littermate, control mice (Fig. 1a). The activation of ROCK1 was restricted to muscle as there was no increase in ROCK1 activity in liver and brown adipose tissue (Fig. 1b). To ascertain the effects of mCaROCK1 on muscle mitochondria, we examined the mitochondrial content in both EDL (Type II fibers) and soleus (Type I fibers) muscles of control and mCaROCK1 mice. The mitochondrial content in soleus muscle of mCaROCK1 mice was significantly decreased compared with the value in soleus muscles of control mice, as evidenced by reduced mitochondrial DNA (mtDNA) copies, and protein levels of SDHA (Succinate Dehydrogenase Complex, Subunit A) and VDAC (Voltage-dependent anion channel) in western blots (Fig. 1c,d). However, we did not find a difference in the mitochondrial content of EDL muscles from mCaROCK1 and control mice, although the expressions of both intact ROCK1 (160 kD) and constitutive active ROCK1 (130 kD) are identical in soleus and EDL muscles (Fig. 1e). Electron microscopy images revealed that mitochondrial sizes in soleus muscles of mCaROCK1 mice were much smaller compared to results in soleus muscle of littermate control mice. In contrast, there was no difference in mitochondrial sizes in EDL muscles of control and mCaROCK1 mice (Supplemental Fig. 2a). These results indicate a differential responses to constitutive active ROCK1 in mitochondrial biogenesis of slow and fast type myofibers, suggesting PGC-1α, a master regulator of mitochondrial biogenesis and myokines expression, might be suppressed by constitutive active ROCK1 in skeletal muscle.

### Muscle-specific ROCK1 activation leads to obesity

**Figure 2:**
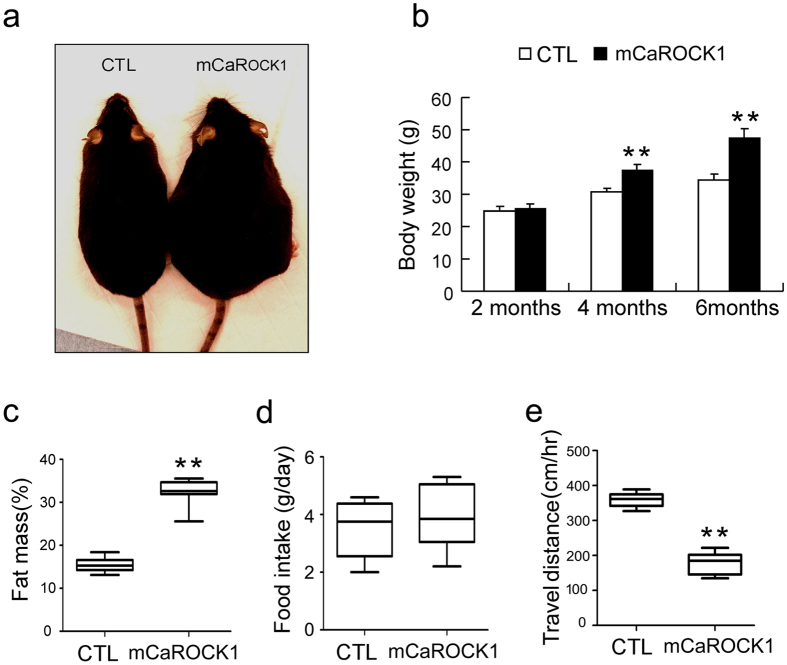
Mice with muscle-specific ROCK1 activation develops obesity. (**a**) Photograph of 6-month-old CTL littermate and mCaROCK1 mice. (**b**) Body weights increased with age in mCaROCK1 mice (mean ± SEM, n = 9, **p < 0.01 vs. CTL). (**c**) Whole body fat mass evaluated by echo-MRI, results were presented as % of bodyweight (mean ± SEM, n = 7, **p < 0.01 vs. CTL). (**d**) Average of 5 days food intake as shown in bar graph (mean ± SEM, n = 7). (**e**) locomotive activity was evaluated by travel distance in control (CTL) and mCaROCK1 mice (mean ± SEM, n = 7, **p < 0.01 vs. CTL).

At 3 month of age, mCaROCK1 mice were obvious obese. After 4 months of age, mCaROCK1 mice weighed ~20% more than littermate, control mice; by 6 months, the mCaROCK1 mice weighted ~35% more than control mice (Fig. 2a,b). Body composition analysis by Echo-MRI showed a significant increase in the whole body fat mass in mCaROCK1 (Fig. 2c) mice. To confirm phonotypic changes, we measured the weight of epididymal fat pad in these mice and found that it was significantly increased in mCaROCK1 mice and compared to results in control mice (1.57 + 0.15 vs. 0.47 + 0.042 g in controls, n = 7). Notably, there were no difference in the food intake of mCaROCK1 and littermate, control mice estimated as the average daily weight of food consumed (Fig. 2d). To determine if the gain of fat in mCaROCK1 mice was associated with differences in physical activity, we monitored the locomotor activity of 6-month-old mCaROCK1 and control mice for 24 hr. Physical activity of mCaROCK1 mice was significantly lower than that of littermate control mice (Fig. 2e). These results indicate that feeding a regular chow led to obesity in mice with muscle-specific ROCK1 activation.

### Muscle-specific ROCK1 activation impairs energy metabolism in mice

**Figure 3:**
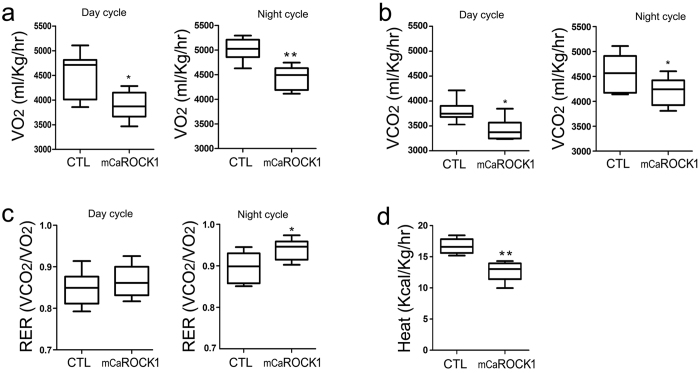
Muscle-specific ROCK1 activation impairs energy metabolism. (**a**,**b**) The day-cycle and night-cycle of oxygen consumption (VO2) and carbon dioxide generation (VCO2) analyzed by indirect calorimetry in CTL and mCaROCK1 mice, the values were normalized with lean body mass. (**c**) Respiratory exchange ratio of mCaROCK1 and CTL mice during day and night cycle. (**d**) mCaROCK1 mice have decreased heat expenditure. All data are presented as the mean ± SEM, n = 7. *p < 0.05 or **p < 0.01 mCaROCK1 vs. CTL.

To determine if muscle-specific ROCK1 activation impacts whole body energy metabolism, we examined levels of oxygen consumed and carbon dioxide produced in 6-month-old mCaROCK1 and control mice. Consistent with the phenotypes observed in mCaROCK1 mice, the mice had a reduced rate of oxygen consumption (VO2) and carbon dioxide production (VCO2) vs. results from littermate, control mice (Fig. 3a,b). Interestingly, the respiratory quotient (RER) was significantly higher in mCaROCK1 mice during the night cycle compared to results of littermate controls (Fig. 3c). These findings indicated there was increased carbohydrate utilization for energy expenditure plus a decrease in the rate of lipid utilization for energy. Heat production by mCaROCK1 mice was significantly lower than in littermate control mice (Fig. 3d). These results suggest that mCaROCK1 mice have a tendency to store lipid. Thus, mCaROCK1 mice have abnormal energy metabolism compared to control mice.

### Muscle-specific ROCK1 activation leads to insulin resistance in mice

**Figure 4:**
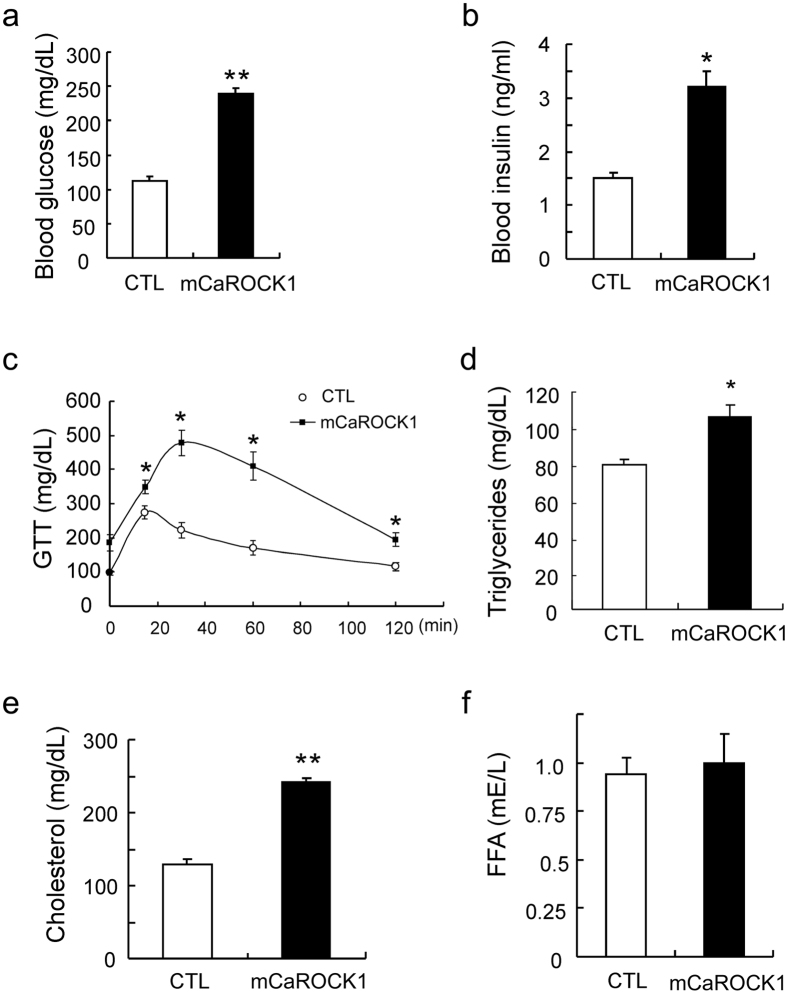
Mice with muscle-specific ROCK1 activation develop insulin resistance. (**a**,**b**) 6 h fasting blood levels of glucose and insulin in CTL and mCaROCK1 mice at age of 6 months. (**c**) Glucose tolerance test (GTT) in CTL and mCaROCK1 mice. (**d–f**) Plasma triglycerides (D), Cholesterol (E), FFA (F) were measured in CTL and mCaROCK1 mice (mean ± SEM, n = 7, *p < 0.05 or **p < 0.01 mCaROCK1 vs. CTL).

Obesity occurs frequently in patients with type-2 diabetes and insulin resistance. To assess insulin resistance in 6-month old mCaROCCK1 mice, we examined blood glucose values and plasma insulin levels. Both were significantly higher in mCaROCK1 mice after a 6 h fast compared to measurements in control mice (Fig. 4a,b). After 6-month of age, mCaROCK1 mice were underwent glucose tolerance test (GTT) which revealed significant impairment of glucose disposal compared to results from control mice (Fig. 4c). mCaROCK1 mice expressed significant increases in plasma triglycerides and cholesterol, but not in free fatty acid (FFA) vs. results in control mice (Fig. 4d–f). Thus, skeletal muscle-specific ROCK1 activation in muscle impairs glucose tolerance and insulin sensitivity in mice.

### Uncoupling protein-1 (UCP-1) expression is decreased in BAT and WAT of mCaROCK1 mice

**Figure 5:**
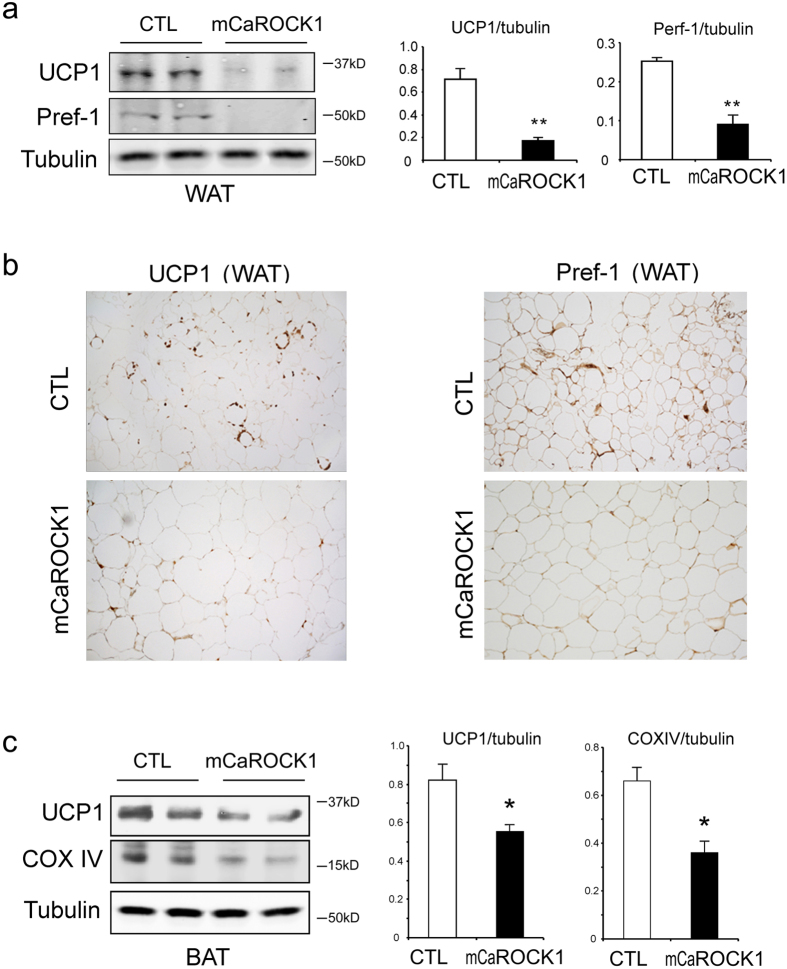
UCP1 expression is decreased in inguinal white adipose tissue (WAT) and brown adipose tissue (BAT) of mCaROCK1 mice. (**a**) Western blot analysis of UCP1 and Pref-1 expressions in WAT of CTL and mCaROCK1 mice (mean ± SEM, n = 5, **p < 0.01 mCaROCK1 vs. CTL). (**b**) Immunohistochemistry of UCP1 (Left panel) and Pref-1(Right panel) in WAT of CTL and mCaROCK1 mice (DAB staining). (**c**) Western blot analysis of UCP1 and Pref-1 expressions in BAT of CTL and mCaR1 mice (mean ± SEM, n = 5, *p < 0.05, mCaROCK1 vs. CTL).

Given the reduced heat production and obesity in mCaROCK1 mice, we analyzed UCP1 expression in inguinal WAT of these mice. UCP1 expression was decreased in inguinal WAT of mCaROCK1 mice (Fig. 5a, middle panel). Since a low level of preadipocyte factor-1(Pref-1, a marker of preadipocytes) is often associated with obesity^29^,^30^, we examined its expression in inguinal WAT in control and mCaROCK1 mice. There was a significant decrease in Pref-1 expression in inguinal WAT of mCaROCK1 mice compared to result in control mice (Fig. 5a); Immunohistochemistry confirmed that UCP1 and Pref-1 positive cells were sharply decreased in inguinal WAT of mCaROCK1 mice (Fig. 5b). There also was found a decrease in UCP1 in BAT of mCaROCk1 mice (Fig. 5c). These results suggest that mCaROCK1 mice have impaired recruitment of “brown-like adipocyte”^31^.

### Irisin expression and secretion are reduced in obese mCaROCK1 mice

**Figure 6:**
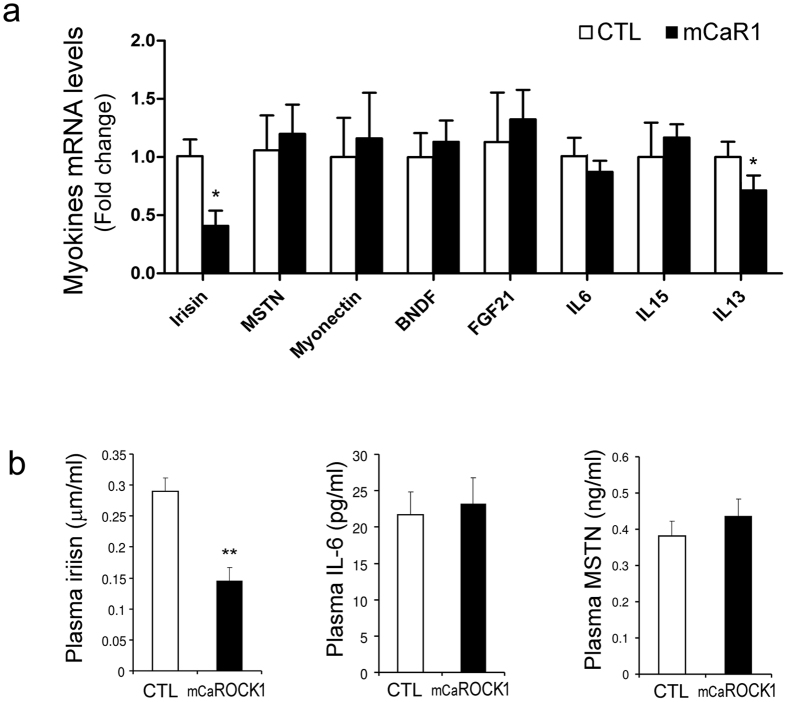
Irisin expression and secretion is decreased in obese mCaROCK1 mice. (**a**) Muscle mRNA level of myockines were examined by quantitative real-time PCR. (**b**) Plasma level of Irisin, IL-6 and Myostatin (MSTN) were measured by Elisa assay. Data are presented as the mean ± SEM, n = 3–5, *p < 0.05, mCaROCK1 vs. CTL.

Since skeletal muscles produce and release multiple myokines which can influence lipid and glucose metabolism, we hypothesized that ROCK1 activation in the muscle of mCaROCK1 mice could stimulate abnormal glucose and lipid metabolism via changes in myokines. Using real-time quantitative PCR, we measured mRNA expressions of the following myokines: IL-6, IL-15, Brain-derived neurotrophic factor (BDNF), myostatin (MSTN), myonectin (CTRP15), fibroblast growth factor 21(FGF21) and the fibronectin type III domain containing 5 protein (FNDC5, precursor of irisin). In muscles of mCaROCK1 mice, we observed a 60% suppression of irisin mRNA expression, a minor, 25% suppression of IL-13 but no change in the other myokines (Fig. 6a). Since myokines are produced by skeletal muscle and released into circulation, we examined plasma levels of myokines including IL-6, myostatin and irisin proteins. As shown in Fig. 6b, only irisin was significantly reduced in the plasma of mCaROCK1 mice; the levels of myostatin and IL-6 were unchanged. These results indicate that muscle-specific activation of ROCK1 suppresses irisin secretion from muscles.

### Synthetic irisin suppresses obesity and improves insulin resistance in mCaROCK1 mice

**Figure 7:**
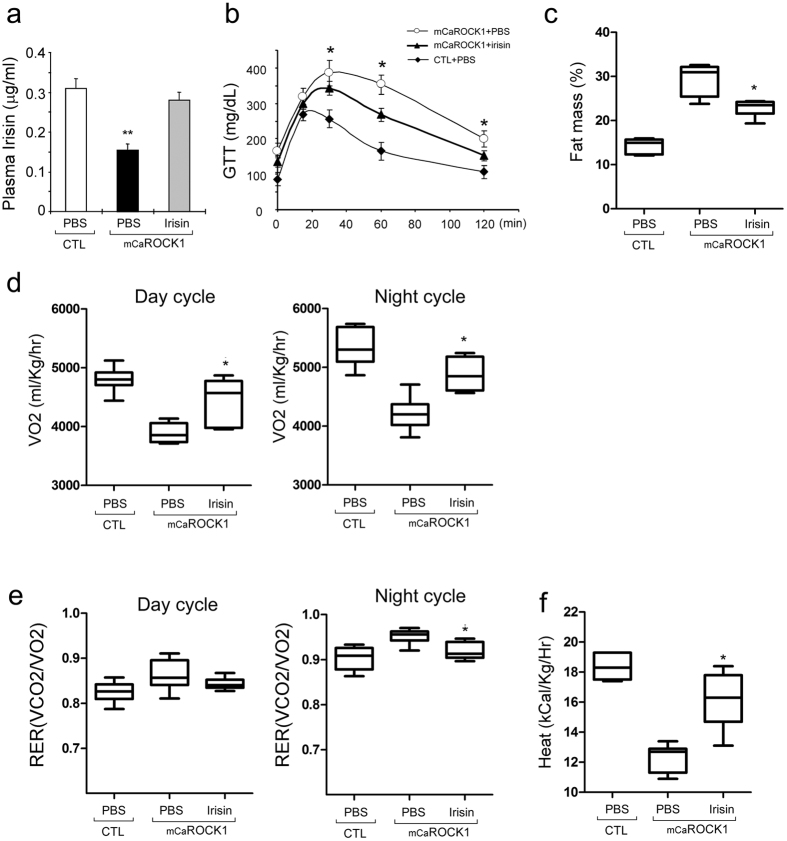
Irisin replenishment improves obesity and insulin resistance in mCaROCK1 mice. (**a**) Blood irisin concentration was determined by ELISA assay. (**b**) Glucose tolerance was improved in mCaR1 mice after irisin treatment. (**c**) After irisin treatment, fat mass was measured by echo-MRI, data is represented as percentage of bodyweight (BW). (**d**) Oxygen consumption (VO2) analyzed by indirect calorimetry in mCaROCK1 mice treated with or without irisin. (**e**) Respiratory exchange ratio of mCaROCK1 and CTL mice. (**f**) Heat generation was measured in mCaR1 mice treated with or without irisin. All data are presented as the mean ± SEM, n = 8, *p < 0.05. mCaROCK1 + Irisin vs. mCaROCK1.

To examine interactions of irisin and adipose tissues in mCaROCK1 mice, we injected synthetic irisin into 4-month-old mCaROCK1 mice for 3 weeks. Plasma irisin in control mice was 0.28 ± 0.018 μg/ml and in mCaROCK1 mice, plasma irisin was 0.152 ± 0.016 μg/ml (Fig. 7a). With a dosage of irisin 100 μg/Kg, ip, daily for 3 weeks, the plasma level of irisin raised to 0.32 ± 0.02 μg/ml in mCaROCK1 mice, close to the level that was comparable to that in control mice (Fig. 7a). Irisin administration partially improved glucose tolerance and decreased the fat mass of mCaROCK1 mice when compared to results of mCaROCK1 mice injected with PBS (Fig. 7b,c). Concomitant with the decrease in fat mass, both day and night cycle oxygen consumption (VO2) was increased by irisin administration (Fig. 7d), and the respiratory quotient (RER) was suppressed during the night cycle in mCaROCK1 mice with irisin injection (Fig. 7e). Heat production was also increased in mCaROCK1 mice treated with irisin compared to results of untreated mCaROCK1 mice (Fig. 7f). We observed no significant changes in physical activity or food intake (Supplemental Fig. 2b and c). Thus, Irisin replenishment over 3 weeks of treatment partially reverses the metabolic defects of mCaROCK1 mice.

### Irisin stimulates UCP1 expression in WAT of mCaROCK1 mice

**Figure 8:**
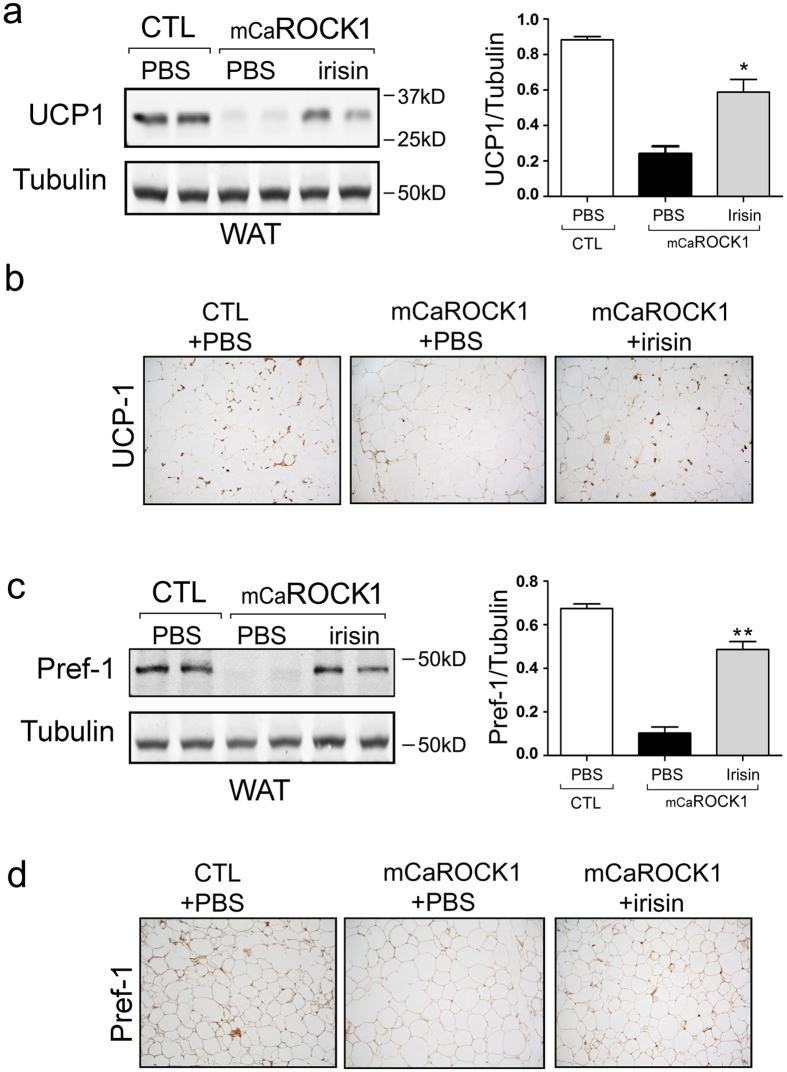
Irisin stimulates UCP1 expression in WAT in mCaROCK1 mice. (**a**) Western blot revealed that UCP1 was significant increase in WAT of mCaROCK1 mice after irisin treatment (mean ± SEM, n = 5, *p < 0.05, **p < 0.01, mCaROCK1 + Irisin vs. mCaROCK1). (**b**) Immunohistochemistry of UCP1 in WAT of mCaROCK1 mice treated with or without irisin (DAB staining). (**c**) Western blot indicated that Perf-1 was significant increase in WAT of mCaROCK1 mice after irisin treatment (mean ± SEM, n = 5, *p < 0.05, **p < 0.01, mCaROCK1 + Irisin vs. mCaROCK1). (**d**) Immunohistochemistry of Perf-1 in WAT of mCaROCK1 mice treated with or without irisin (DAB staining).

To understand how irisin administration might suppress adipose tissue accumulation, we examined the expression of UCP1 and Pref-1 in inguinal WAT of mCaROCK1 mice. UCP1 expression in inguinal WAT of PBS-treated mCaROCK1 mice was significantly lower than results from littermate, control mice. After 3 weeks of irisin injection, the UCP1 expression in WAT was significantly increased in mCaRCOK1 mice (Fig. 8a), Immunohistochemistry confirmed the increase in UCP1 positive cells in WAT of mCaROCK1 mice treated with irisin (Fig. 8b). Similarly, Pref-1 expression was significantly increased in WAT of mCaROCK1 mice treated with irisin (Fig. 8c,d). Although infusion of irisin partially reverses the phenotypic changes in mCaROCK1 mice, the results are consistent with the hypothesis that irisin stimulates the conversion of preadipocytes into brown-like adipocytes in inguinal WAT and hence increasing thermogenesis in mCaROCK1 mice.

### ROCK1 activation suppresses Irisin expression through down-regulation of PGC1a in muscle cells

**Figure 9:**
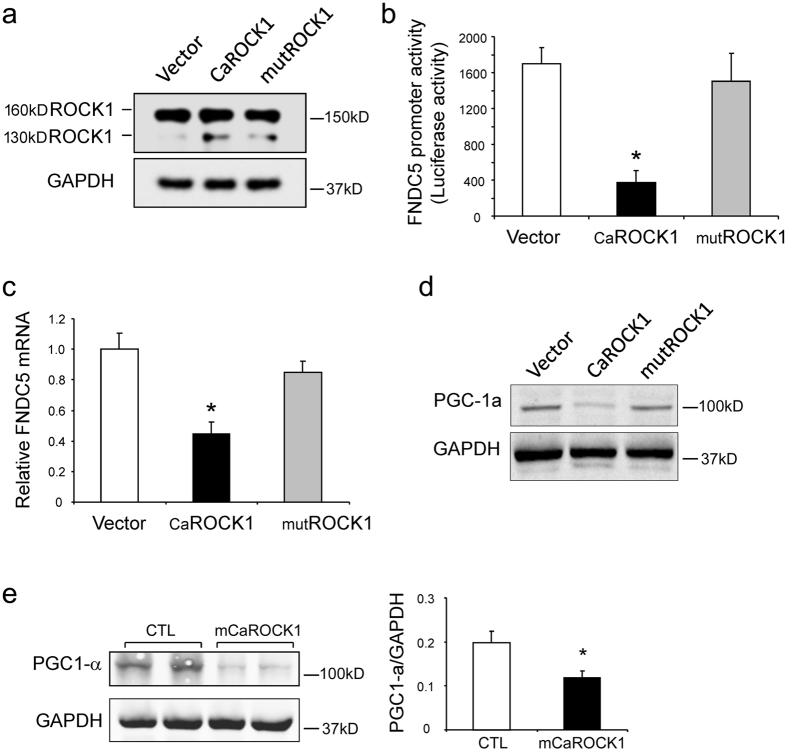
Constitutive ROCK1 activation suppresses Irisin expression via down-regulation of PGC1a. (**a**) C_2_C_12_ cells were transfected with control (empty vector), or overexpression vector encoding constitutive activation ROCK1 (CaROCK1, 130 kD), or kinase mutant ROCK1 (mutROCK1, 130 kD). After 24 h, endogenous ROCK1 (total ROCK1) and CaROCK1 or mutROCK1 were accessed by western blot. (**b**) PLG3 LUC reporter plasmid containing FNDC5 (precursor of irisin) promoter were co-transfected with control vector or CaROCK1 or mutROCK1 into C_2_C_12_, LUC activities were measured after 24 h of transfection (mean ± SEM, n = 5, *p < 0.05, vs. Vector). (**c**) mRNA levels were accessed with qRT-PCR in C_2_C_12_ cells transfected with empty vector; CaROCK1 or mutROCK1 (mean ± SEM, n = 3, *p < 0.05, vs. Vector). (**d**) PGC-1a level were examined by western blot in C_2_C_12_ cells transfected with plasmids as indicated. (**e**) Western blot analysis of PGC-1a in muscles of CTL littermates and mCaROCK1 mice (mean ± SEM, n = 5, *p < 0.05, vs. CTL).

We also examined the mechanism by which ROCK1 activation suppresses irisin expression. We co-transfected plasmids expressing constitutive active ROCK1 (caROCK1) and a LUC3 reporter construct containing the FNDC5 promoter into C_2_C_12_ myoblasts. CaROCK1 expression led to an increase in the 130 kD, active ROCK1 (Fig. 9a) level. The increase in ROCK1 activity repressed the FNDC5 promoter activity in C_2_C_12_ cells (Fig. 9b); there also was a decrease in the FNDC5 mRNA expression (Fig. 9c). Importantly, expression of a kinase mutated CaROCK1 (mutROCK1) did not suppress FNDC5 promoter activity or the mRNA of FNDC5. Because PGC-1αcan stimulate irisin expression, we examined PGC-1a expression in C_2_C_12_ cells transfected with caROCK1 or mutROCK1. As shown in Fig. 9d, overexpression of caROCK1 suppressed PGC-1α protein in muscle cells while overexpression of mutROCK1 did not change PGC-1a expression, indicating that constitutively active ROCK1 in muscle cells block irisin expression by down-regulating PGC-1α. In support of this conclusion, we found that PGC-1a expression in muscles of mCaROCK1 mice was reduced compared to the results in control littermates (Fig. 9e). Therefore, constitutive ROCK1 activation suppresses PGC-1α resulting in reduced irisin expression.

## Discussion

Irisin reportedly recruits brown adipocytes into white adipose tissue, a phenomenon known as “browning”^16^. This is relevant because browning of WAT in rodents can lead to decreased adipogenesis and improved insulin sensitivity^15^,^32^,^33^. Interestingly, the browning response has been noted with an increased circulating irisin which occurs after exercise^16^. We investigated whether a lower level of irisin occurring in rodents at rest would change the metabolism of adipose tissues. We studied mice with muscle-specific ROCK1 activity (mCaROCK1) because these mice generally develop insulin resistance and obesity. These phenotypes also stimulate us to test if irisin administration corrects insulin resistance and obesity in mCaRCOK1 mice. When we examined relationships among plasma and muscle levels of irisin plus obesity and insulin resistance, we found that the plasma irisin in resting mCaROCK1 mice was significantly (p < 0.01) lower vs. plasma irisin levels of control mice. Moreover, administration of synthetic irisin to mCaROCK1 mice largely corrected their obesity and insulin resistance. These results are compatible with the hypothesis that a low level of circulating irisin contributes to the development of obesity.

Reduced physical activity and insulin resistance frequently develop in patients with chronic diseases resulting in insulin resistance^9^,^34^. For example, patients with chronic kidney disease (CKD) or diabetes as well as sedentary adults develop insulin resistance and these clinical conditions are associated with down-regulation of irisin^35^,^36^,^37^. Since irisin is an exercise-induced hormone, it could be the mediator that links reduced physical activity with the development of insulin resistance. Consistent with these reports, we found that decreased physical activity is associated with low plasma level of irisin in mCaROCK1 mice. Importantly, treatment of mCaROCK1 mice with recombinant irisin led to correction of the glucose tolerance test despite the fact that there was no change in the physical activity of the mice. These results demonstrate that a low level of irisin can correct the development of insulin resistance.

ROCK-1 is activated by small GTPases (inducible activation) or by caspase-3 cleavage (constitutive activation). ROCK1 activity stimulates cellular processes that include mitochondrial fission and autophagy as well as pathophysiological processes that include obesity^38^, diabetic nephropathy^18^ and atherosclerosis^39^. Interestingly, in type 1 myofibers present in mCaROCK1 mice, we found a decrease in the mitochondrial content. Potentially, this abnormality arises from a lower level of PGC-1a, the master regulator of mitochondria (Fig. 9). Another potential mechanism for loss of mitochondria would be activation of mitochondrial fission with mitophagy^18^,^19^. However, we have not evaluated these mechanisms for reduced mitochondria.

ROCK1 reportedly adversely influences insulin sensitivity and adipogenesis. For example, a chemical ROCK1 inhibitor can cause insulin resistance plus impaired intracellular insulin signaling in mouse muscles^40^. Conversely, ROCK1 deletion in adipocytes improves insulin sensitivity ad obesity in mice fed a high-fat diet^38^. These reports are emphasized because they stimulated us to examine how muscle-specific ROCK1 activation influences insulin sensitivity and the development of obesity.

We generated a mouse with muscle-specific, constitutively active ROCK1 and this mouse develops obesity and insulin resistance, suggesting two separate mechanisms are responsible for insulin resistance: 1) ROCK1 is activated by Rho A or Rho E causing PKC activation and suppressed glycogenesis^41^. 2) When ROCK1 is constitutively activated (by caspase-3 cleavage)^22^,^28^, it will suppress irisin expression leading to adipogenesis and insulin resistance. Thus, activation of ROCK1 by Rho A or Rho E seems to be important for the muscle glycogenesis, whereas constitutive activation of ROCK1 suppresses irisin production to impair impairing glucose uptake and energy expenditure.

How dose muscle-specific ROCK1 activation decrease plasma Irisin? Irisin secretion is stimulated by exercise or in response to PGC-1a expression^16^,^42^. Thus, our results may reflect that mCaROCK1 mice are more sedentary than control mice leading to suppression of irisin in muscle. It is also possible that ROCK1 regulates transcription or secretion of irisin, because the mRNA level of irisin was decreased in muscles of mCaROCK1 mice. Alternatively, Bostrom *et al*. reported that PGC-1α, a mitochondrial biogenesis regulator, regulates irisin expression at the transcriptional level^16^. In contrast, ROCK1 has detrimental effects on mitochondrial function and cellular energy metabolism in diabetes. For example, it is reported that ROCK1 activation causes mitochondrial fission and mitochondrial dysfunction in endothelial cells^18^. Possibly, the adverse influence of ROCK1 on PGC-1a might reduce irisin production in mCaROCK1 mice. In support this hypothesis, we find that constitutive ROCK1 activation suppresses PGC-1a expression and reduced FNDC5 promoter activity and its mRNA expression. The mechanism of ROCK1 down-regulating PGC-1a remains to be fully elucidated.

In conclusion, we have demonstrated that muscle-specific ROCK1 activation suppresses the production of circulating irisin. Low levels of irisin leads to a decrease in brown-like adipocytes found in inguinal WAT and hence, reduced heat production. Thus, our results highlight irisin mediating muscle-adipose tissue communication; reduced irisin expression contributes to the development of obesity and insulin resistance in mCaROCK1 mice.

## Additional Information

**How to cite this article**: Zhou, X. *et al*. ROCK1 reduces mitochondrial content and irisin production in muscle suppressing adipocyte browning and impairing insulin sensitivity. *Sci. Rep*. **6**, 29669; doi: 10.1038/srep29669 (2016).

## Supplementary Material

Supplementary Information
