# Supplementary material for: ROCK1 reduces mitochondrial content and irisin production in muscle suppressing adipocyte browning and impairing insulin sensitivity
Source: Sci Rep. 2016 Jul 14;6:29669. doi: 10.1038/srep29669 (PMC4944137; doi:10.1038/srep29669)
Supplement: Supplementary Information [file srep29669-s1.doc]

## Supplemental information

## ROCK1 reduces mitochondrial content and irisin production in muscle suppressing

## adipocyte browning and impairing insulin sensitivity

## Running title: a low Irisin links to obesity

Xiaoshuang ZhouP ^1^P, Rongshan Li P^1^P, Xinyan LiuP^2^P, Lihua WangP^2^P, Peng HuiP ^3^P, Lawrence ChanP^4^P, Pradip K. SahaP^4^P, Zhaoyong HuP^5^P

^1^ Nephrology Division, Shanxi Province People's Hospital of Shanxi Medical University, Taiyuan, China

^2^ Nephrology Division, Second Hospital of Shanxi Medical University, Taiyuan, China

^3^ Nephrology Division, The third affiliated hospital of Sun Yat-sen University, Guangzhou, China

^4^ ­­Endocrinology Division, Department of Medicine and

^5^ Nephrology Division, Department of Medicine, Baylor College of Medicine, Houston, Texas, USA

**
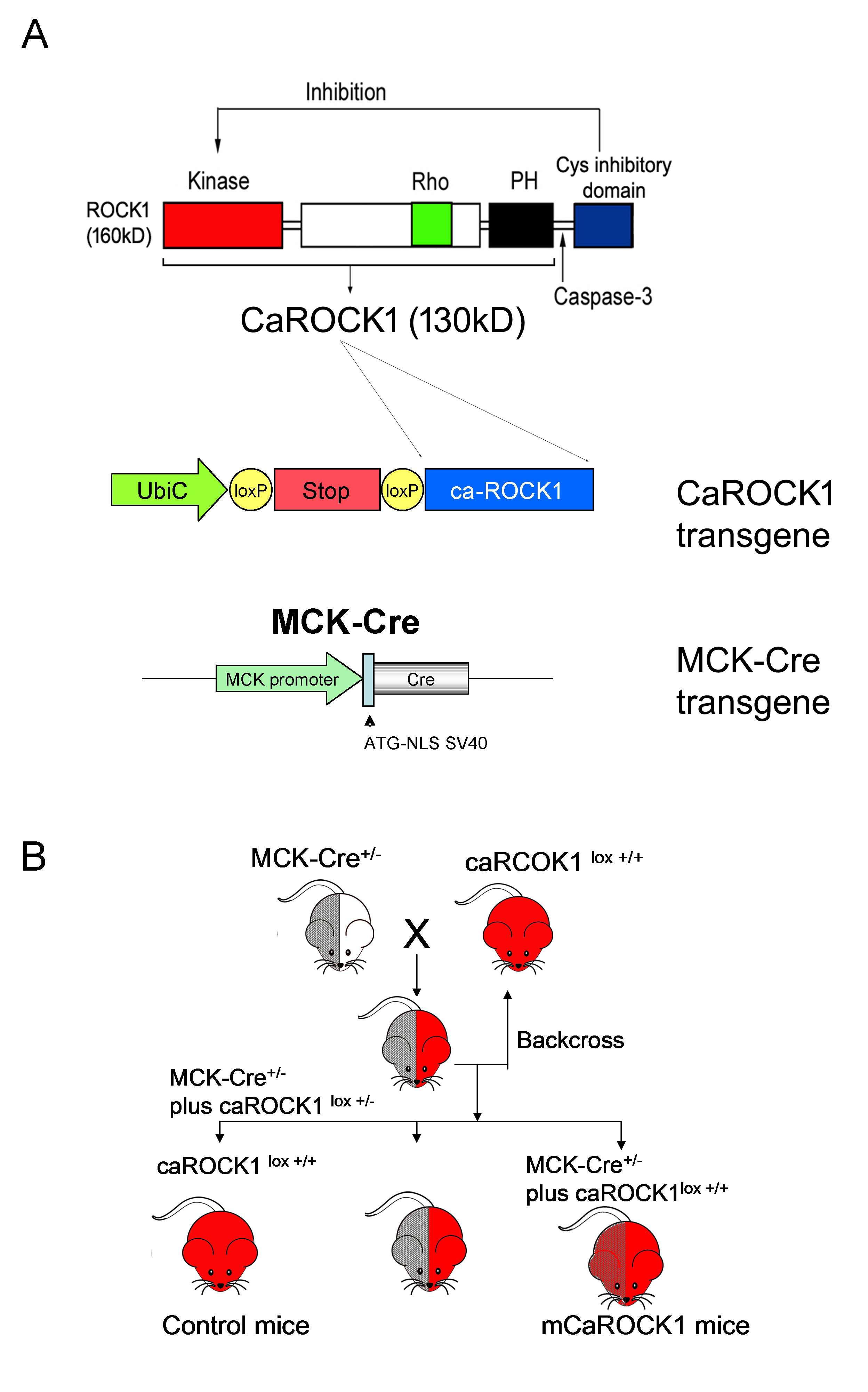
**

**Supplemental Figure 1. Mice with muscle-specific constitutive ROCK1 activation (mCaROCK1)**

**A:** The schematic diagram of constitutively active ROCK1 transgene (upper) and MCK-Cre (lower) transgene. Intact ROCK1 (molecular weight ~160 kD) is consist of kinase domain, RhoA binding domain, PH domain and a inhibitory Cys domain. Caspase-3 can cleave ROCK1 yielding a 130kD, constitutively activate form of ROCK1 (CaROCK1). **B**: Schematic diagram of mCaROCK1 mice breeding strategy

**
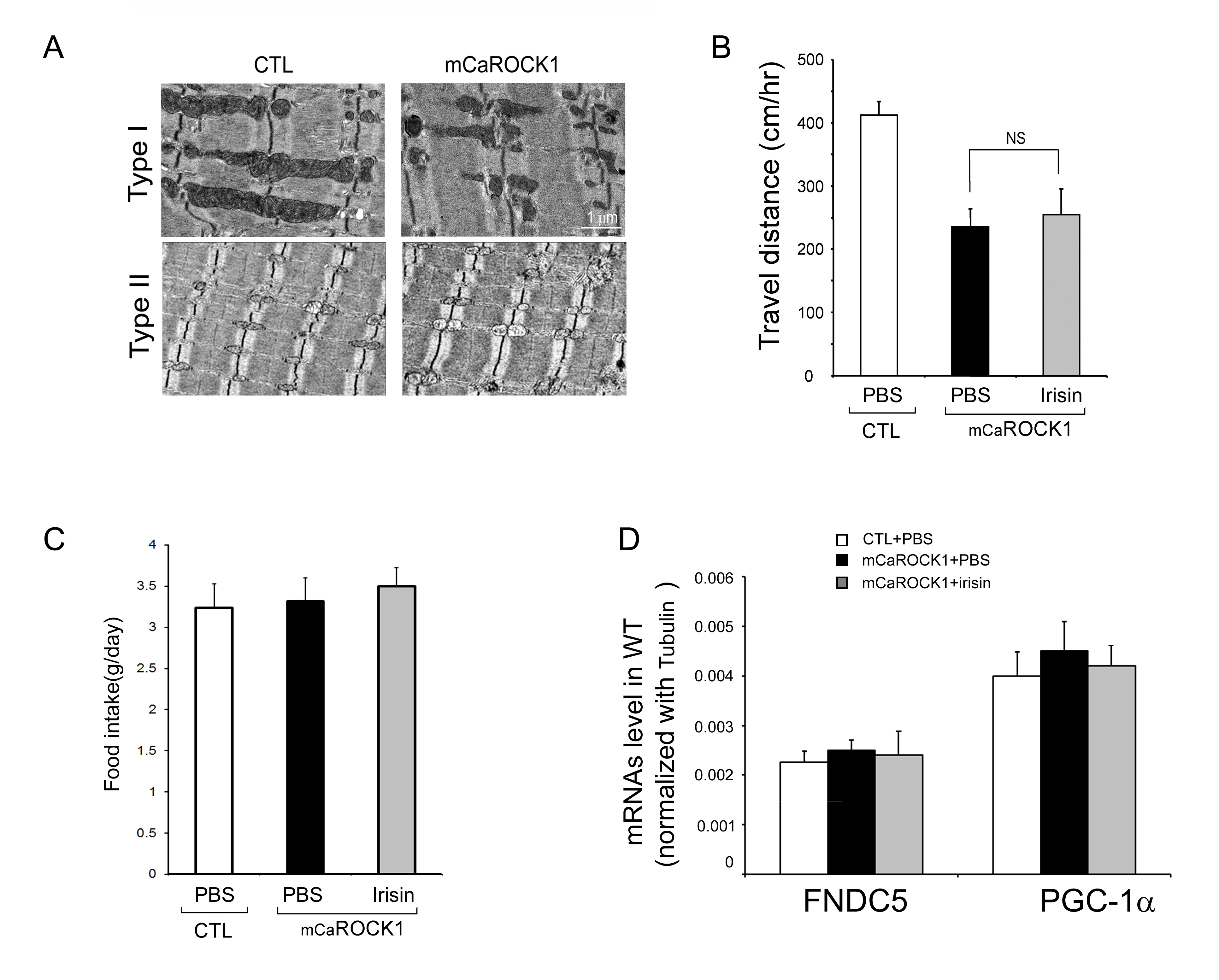
**

**Supplemental Figure 2.**

**A:** Representative electronic microscopy images of intramuscular mitochondria in Sol and EDL muscles of in Control and mCaROCK1 mice.

**B**: locomotive activity was evaluated by travel distance in control (CTL) and mCaROCK1 mice treated with or without irisin. Data are presented as the mean± SEM, n=8, *p<0.05. mCaROCK1+Irisin vs. mCaROCK1.

**C:** Food consumed in CTL and mCaROCK1 mice treated with or without irisin. Data are presented as the mean± SEM, n=8, *p<0.05. mCaROCK1+Irisin vs. mCaROCK1.

**D**: FNDC5 and PGC-1α mRNA expressions in WAT CTL and mCaROCK1 mice treated with or without irisin (mean± SEM, n=5).

**Supplemental Figure 3**

**
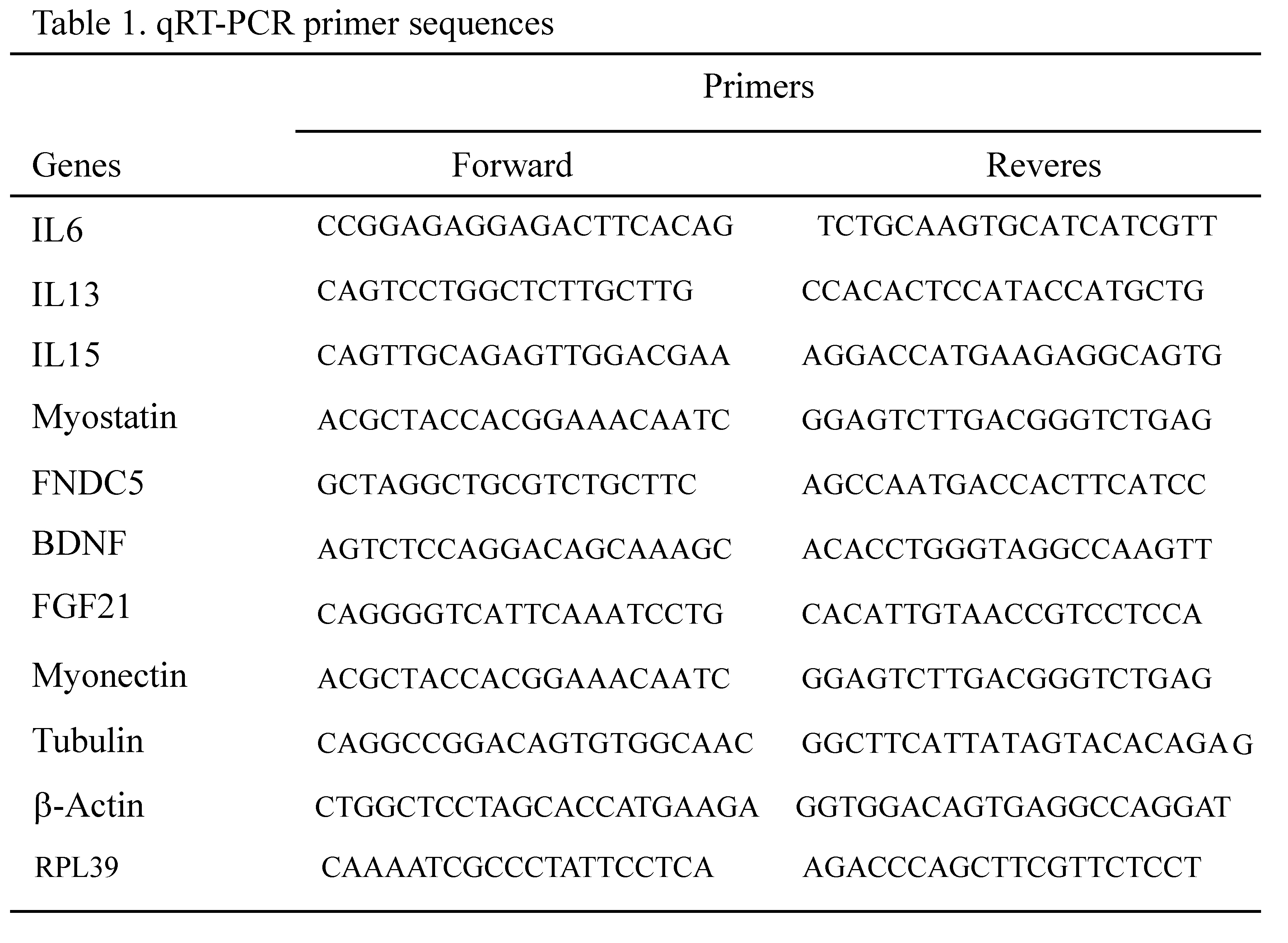
**
